# Intravitreal Steroids for the Treatment of Retinal Diseases

**DOI:** 10.1155/2014/989501

**Published:** 2014-01-08

**Authors:** Valentina Sarao, Daniele Veritti, Francesco Boscia, Paolo Lanzetta

**Affiliations:** ^1^Department of Ophthalmology, University of Udine, Piazza Santa Maria della Misericordia, 33100 Udine, Italy; ^2^Department of Ophthalmology, University of Sassari, Piazza D'Armi, 07100 Sassari, Italy

## Abstract

Diabetic macular edema (DME), pseudophakic cystoid macular edema (CME), age-related macular degeneration (AMD), retinal vascular occlusion (RVO), and uveitis are ocular conditions related to severe visual impairment worldwide. Corticosteroids have been widely used in the treatment of these retinal diseases, due to their well-known antiangiogenic, antiedematous, and anti-inflammatory properties. Intravitreal steroids have emerged as novel and essential tools in the ophthalmologist's armamentarium, allowing for maximization of drug efficacy and limited risk of systemic side effects. Recent advances in ocular drug delivery methods led to the development of intraocular implants, which help to provide prolonged treatment with controlled drug release. Moreover, they may add some potential advantages over traditional intraocular injections by delivering certain rates of drug directly to the site of action, amplifying the drug's half-life, contributing in the minimization of peak plasma levels of the drug, and avoiding the side effects associated with repeated intravitreal injections. The purpose of this review is to provide an update on the use of intravitreal steroids as a treatment option for a variety of retinal diseases and to review the current literature considering their properties, safety, and adverse events.

## 1. Introduction

The use of corticosteroids for the treatment of ocular inflammatory diseases was first described in the early 1950s [1]. Corticosteroids have anti-inflammatory, antiangiogenic, and antipermeability properties that make them an attractive therapeutic option for a variety of posterior segment diseases. The rationale for using a steroidal drug for the treatment of edematous and proliferative diseases is that abnormal proliferation of cells is often associated with and trigged by inflammation. Moreover, intraretinal accumulation of fluid is usually accompanied by a blood-retinal barrier dysfunction that can be restored with steroid therapy. The principal effects of steroids are thought to be stabilization of the blood-retinal barrier (BRB), reduction of exudation, and downregulation of inflammatory stimuli, but the exact mechanisms remain unknown. Steroids are thought to act by the induction of proteins called lipocortins, in particular phospholipase A2. These proteins reduce leukocyte chemotaxis, control biosynthesis, and inhibit the release of arachidonic acid from the phospholipid membrane, which is one of the most important common precursors of potent inflammatory cell mediators such as prostaglandins and leukotrienes. Based on experimental studies, corticosteroids have been shown to control gene expression of inflammatory mediators. This regulation influences the expression of vascular endothelial growth factor (VEGF), inhibits pro-inflammatory genes such as tumor necrosis factor-alpha (TNF-*α*) and other inflammatory chemokines, and induces the expression of anti-inflammatory factors such as pigment-derived growth factor (PEDF) [2–4]. Additionally, steroids seem to reduce the expression of matrix metalloproteinases (MMPs) and to downregulate intercellular adhesion molecule 1 (ICAM-1) on choroidal endothelial cells [5–11]. Several routes of administration have been considered for the treatment of various ocular diseases. Oral dosing, unfortunately, causes a spectrum of systemic side effects, including osteoporosis, cushingoid state, adrenal suppression, and exacerbation of diabetes [12, 13]. Topical steroids have not been shown to penetrate adequately to the posterior segment [14]. Geroski and Edelhauser reported that therapeutic doses of steroids could reach the posterior segment via transscleral absorption with periocular administration [15]. Thus, other routes of administration, such as subconjunctival, subtenon, and posterior juxtascleral infusions, have been studied [16–18]. Periocular delivery of steroids has offered for many years a valid compromise between better penetration and lack of systemic side effects. However, peribulbar injections seem to result in lower morphological and functional outcomes as compared with those reported with the use of intravitreal administration [19–22]. But, two interventional case series have demonstrated that posterior juxtascleral infusion of a viscoelastic formulation of triamcinolone acetonide is an effective treatment for diffuse diabetic macular edema (DME) unresponsive to laser photocoagulation [23, 24].

Based on experimental studies, clinical observations, and pathogenic considerations, Robert Machemer, among others, suggested the intravitreal delivery of steroids to locally suppress intraocular inflammation, proliferation of cells, and neovascularization [25]. Intravitreal delivery of corticosteroids has allowed many posterior segment diseases to be locally treated without the adverse systemic side effects. Intravitreal steroids have been widely studied in many randomized clinical trials, demonstrating significant improvements both in morphological and functional outcomes in many posterior segment diseases [26–28]. Intravitreal therapy also allows for the steroid to bypass the BRB, leading to a more concentrated dose of steroid for a prolonged period of time. Delivery of steroids to the vitreous cavity can be achieved via direct injection through the pars plana, introduction of a sustained-release or biodegradable implants, or injection of conjugate compounds. Several intravitreal biodegradable and nondegradable steroid releasing implants have been designed to provide long-term drug delivery to the macular region. Different steroid molecules have varying potencies and toxicities. There are several ways to distinguish among the steroids used in ophthalmology, including chemical structure, anti-inflammatory potency, ability to translocate the glucocorticoid receptor complex to the nucleus, ability to transactivate or transrepress ligand-dependent gene sets and biologic responses, neuroprotection of the photoreceptors/retinal pigment epithelium, and direct cytotoxic effects [29]. These differences may help to explain the differences among steroids in their safety and efficacy for the treatment of retinal disease. The purpose of this paper is to review the current status of intravitreal steroidal drugs, including triamcinolone acetonide, biodegradable dexamethasone implant, and nondegradable fluocinolone acetonide implant in the treatment of various retinal diseases such as diabetic macular edema (DME), central and branch retinal vein occlusion (CRVO and BRVO), neovascular age-related macular degeneration (AMD), pseudophakic cystoid macular edema (CME), and macular edema secondary to uveitis.

## 2. Triamcinolone Acetonide

Triamcinolone acetonide (TA) is a synthetic steroid of the glucocorticoid family with a fluorine in the ninth position [30]. It is commercially available as an ester and represents one of the most commonly used steroid agents for the treatment of several retinal conditions [31]. TA has an anti-inflammatory potency five times higher than hydrocortisone with a tenth of the sodium-retaining potency. It appears as a white- to cream-colored crystalline powder and it is practically insoluble in water and very soluble in alcohol [14]. The decreased water solubility accounts for its prolonged duration of action. It has been observed that adequate concentrations of TA could provide therapeutic effects for approximately three months after 4 mg intravitreal TA injection [32]. Maximum effect duration of 140 days has been suggested [33].

The current commercial preparations of TA include products that received dermatologic and orthopedic indications and are considered off-label for the intraocular use, products registered as devices for assisting the visualization of the vitreous during vitreoretinal procedures, and products that are registered for intraocular use in uveitis, and other ocular inflammatory conditions. Kenalog-40 (40 mg/mL, Bristol-Myers Squibb, NJ) is the most commonly used intraocular steroid and has been widely utilized as intravitreal injections since 2004 for the treatment of several retinal diseases. This formulation is US Food and Drug Administration (FDA)-approved only for intramuscular and intra-articular use and is currently employed off-label for intraocular injections. TrivarisTM (80 mg/mL, Allergan Inc., Irvine, CA) and Triesence (40 mg/mL, Alcon Inc., Fort Worth, TX) are preservative-free brands of TA recently FDA approved for ophthalmic use in the treatment of sympathetic ophthalmia, temporal arteritis, uveitis, and other ocular inflammatory diseases, unresponsive to topical corticosteroids. Vitreal S (Sooft s.p.a., Fermo, Italy) is a medical device used in endocular surgery to stain the vitreous during vitrectomy and it is not registered as drug for intraocular use. There are some issues regarding the formulation of TA used for intraocular administration. A previous phase-contrast microscopy study showed a notable difference of crystal size depending upon the drug formulation [34]. Very large and irregular crystals, with a significant heterogeneity in crystal size, were occasionally found in the off-label, commercially available, benzyl-alcohol-preserved TA, whereas the crystals of a preservative-free in-label, commercially available, TA suspension appeared to be relatively uniform in size. These morphologic aspects may have a significant impact on the half-life of the drug both in vivo and in vitro. This hypothesis is based on the fact that smaller crystals have a superior surface-area-to-volume ratio, allowing them to be dissolved more rapidly. The formulations containing crystals that widely vary in size and, thus, including larger crystals may theoretically generate a wider time–drug concentration curve because of their slower dissolution rate. Different TA formulations show variance in reducing the endothelial cell proliferation.

The appropriate dose of intravitreal TA remains a subject of debate. Both Audren et al. and Hauser et al. showed that the use of a 4 mg dose of intravitreal TA does not have enough advantages over the lower 1 mg or 2 mg dose [35, 36]. However, Lam et al. published a comparison between 4 mg and 8 mg doses and showed that the higher dose had a more sustained effect on both visual acuity and central macular thickness, although with a trend to more ocular complications [37]. By using a dose of about 20 mg of TA, the increase in visual acuity was mostly marked during the first three and six months after injection and was observable for a period of about six to nine months. Differently, by using a dose of 4 mg, the duration in the reduction of macular thickness as measured by optical coherence tomography (OCT) was less than six months [38].

Based on several studies, intravitreal administration of triamcinolone acetonide (TA) has provided promising results for the treatment of disorders associated with an abnormal endothelial cell proliferation and conditions complicated by intraretinal and subretinal fluid accumulation. The anti-inflammatory, angiostatic, and antipermeability properties of TA have gained interest in chronic retinal diseases, such as proliferative diabetic retinopathy [39], DME [40, 41], exudative AMD [42–44], presumed ocular histoplasmosis syndrome [45], CRVO [46], BRVO [47], neovascular glaucoma [48], proliferative vitreoretinopathy [49], persistent pseudophakic CME [50], perifoveal telangiectasias [51], sympathetic ophthalmia [52], ischemic ophthalmopathy [53], exudative retinal detachment [54], radiation induced macular edema [55], macular edema due to retinitis pigmentosa [56], Vogt-Koyanagi-Harada syndrome [57], and chronic uveitis [58].

### 2.1. Diabetic Macular Edema

Intravitreal TA has been widely studied in many randomized clinical trials on DME demonstrating significant improvements both in morphological and functional outcomes [40, 41, 59–61]. Focal and grid laser photocoagulation have been considered the standard of care for the treatment of DME for many years. However, a substantial group of patients are unresponsive to laser therapy and fail to improve after photocoagulation. It has been reported that three years after initial grid treatment, visual acuity improved in 14.5% of the eyes, did not change in 60.9%, and decreased in 24.6% of patients with DME [59]. Therefore, TA has been tested for the treatment of DME, either naïve or diffuse and refractory to laser therapy. In most cases, TA has been administered intravitreally.

A carefully designed prospective randomized trial conducted by the Diabetic Retinopathy Clinical Research Network (DRCR.net) investigated the efficacy and safety of 1-mg and 4-mg doses of preservative-free intravitreal TA (Trivaris) in comparison with focal or grid laser photocoagulation [60]. In the DRCR.net study, 840 study eyes affected by DME were randomized to either focal or grid laser photocoagulation (*n* = 330), 1 mg TA (*n* = 256) or 4 mg TA (*n* = 254). At 36 months, the mean change in the visual acuity from baseline was +5 letters in the laser group and 0 letters in both TA groups. A worsening in visual acuity of three or more lines occurred in 8%, 17%, and 16% of eyes, respectively, and an improvement in visual acuity by three or more lines occurred in 26%, 20%, and 21% of eyes, respectively. Mean (±SD) reductions in central macular thickness were 175 ± 149 *μ*m in the laser group, 124 ± 184 *μ*m in the 1 mg TA group, and 126 ± 159 *μ*m in the 4 mg TA group. The mean number of treatments at the end of the follow-up was 3.1 for the laser group, 4.2 for the 1 mg, and 4.1 for the 4 mg TA groups. At the four-month visit, mean visual acuity improvement was higher in the 4 mg TA group (4 ± 12 letters improvement) than in either the laser group (0 ± 13 letters change) or the 1 mg TA group (0 ± 13 letters change). By 12 months, there were no significant differences among groups in mean visual acuity. Therefore, in this study, photocoagulation was shown to be more effective over time and had fewer side effects than TA. This was considered in support of focal/grid photocoagulation. However, it must be noted that during the 36 months of follow-up, patients received only four treatments with intravitreal TA, which is a low reinjection rate based on pharmacokinetic data. Recently, a new, large, randomized DRCR.net study investigated the efficacy of intravitreal TA in combination with laser photocoagulation in comparison with intravitreal ranibizumab with prompt or deferred laser photocoagulation or laser photocoagulation alone. At 2-year visit, mean change (±SD) in the visual acuity letter score from baseline was +7 ± 13 in the ranibizumab + prompt laser group, +9 ± 14 in ranibizumab + deferred laser group, +2 ± 19 in the TA + prompt laser group, and +3 ± 15 the sham + prompt laser group. Compared with the sham + prompt laser group, the difference in mean change in the visual acuity letter score from baseline was 3.7 letters greater in the ranibizumab + prompt laser group (*P* = 0.03), 5.8 letters greater in the ranibizumab + deferred laser group (*P* < 0.01), and 1.5 letters worse in the TA + prompt laser group (*P* = 0.35). A worsening of visual acuity of three or more lines occurred in 10%, 4%, 2%, and 13% of eyes, respectively, and an improvement in visual acuity by three or more lines occurred in 18%, 29%, 28%, and 22% of eyes, respectively. The mean change (*μ*m ± SD) in central retinal thickness from baseline was −141 ± 155 in the ranibizumab + prompt laser group, −150 ± 143 in ranibizumab + deferred laser group, −107 ± 145 in the TA + prompt laser group, and −138 ± 149 the sham + prompt laser group. Compared with the sham + prompt laser group, the difference in mean change in central macular thickness from baseline was 31 *μ*m worse in the ranibizumab + prompt laser group (*P* = 0.03), 28 *μ*m worse in the ranibizumab + deferred laser group (*P* = 0.01), and 10 *μ*m worse in the TA + prompt laser group (*P* = 0.37). These results showed that intravitreal ranibizumab with prompt or deferred laser is more effective than prompt laser alone or intravitreal TA combined with laser for the treatment of DME involving the central macula. Among the eyes that were pseudophakic at baseline, the mean change (±SD) in the visual acuity letter score from baseline was +5 ± 17 in the ranibizumab + prompt laser group, +9 ± 17 in ranibizumab + deferred laser group, +8 ± 13 in the TA + prompt laser group, and +5 ± 15 the sham + prompt laser group. The difference in mean change in visual acuity letter score from baseline to the two-year visit was 1.6 letters greater in the TA + prompt laser group compared with the sham + prompt laser group and was similar to difference in outcomes between the ranibizumab + prompt laser group (+0.5 letters) and the ranibizumab + deferred laser group (+3.5 letters) compared with the sham + prompt laser group. Cataract surgery was required in 12% of phakic eyes in the sham + prompt laser and in the ranibizumab + prompt laser groups, in 13% of phakic eyes in the ranibizumab + deferred laser group, and in 55% of patients of the TA + laser group. An intraocular pressure (IOP)-lowering medication was required in 5% of eyes in the sham + prompt laser and ranibizumab + prompt laser groups, in 3% of eyes in the ranibizumab + deferred laser group, and in 28% of patients of the TA + laser group [61]. Other studies demonstrated promising results of combination therapy with intravitreal injection of TA and laser photocoagulation for the treatment of proliferative diabetic retinopathy (PDR) with clinically significant macular edema (CSME) [62–67]. In a 12-month randomized clinical trial conducted by Maia et al., 44 eyes with PDR and CSME were enrolled and randomized to treatment with combined 4 mg of intravitreal TA and laser photocoagulation (*n* = 22) or to laser photocoagulation alone (*n* = 22). Mean best correct visual acuity (BCVA) improved significantly (*P* < 0.001) in the TA and laser group compared with the laser alone group at all study follow-up visits. An improvement of two or more Early Treatment Diabetic Retinopathy Study (ETDRS) lines was observed in 63.1% and 10.5% of eyes, respectively (*P* < 0.001). A significant decrease in mean central macular thickness occurred in the TA and laser group when compared with the laser alone group at all study follow-up intervals (*P* < 0.001). At 12 months, mean (±SD) reductions in central macular thickness were 123 ± 68 *μ*m and 65 ± 51 *μ*m, respectively (*P* < 0.001) [67]. Several other studies reported positive results of intravitreal TA in refractory DME [68–71]. In a six-month prospective, placebo-controlled, randomized clinical trial conducted by Jonas et al., 40 eyes with persistent DME were enrolled and randomized to treatment with 20 mg TA (*n* = 28) or to placebo injection (*n* = 12). Visual acuity increased significantly (*P* < 0.001) in the TA group by 3.4 ETDRS lines. In the placebo group, visual acuity did not change significantly (*P* = 0.07) during the six months. At the end of the follow-up period, 48% in the TA group improved by at least two ETDRS lines compared with 0% eyes in the placebo group [69]. Recently, Gillies et al. reported the longest-term data available concerning the outcomes of intravitreal injection of TA. This was a five-year prospective, double-masked, randomized clinical trial of 4 mg dose of preservative-free intravitreal TA in comparison with placebo. In this study, 67 study eyes with refractory DME were randomized to receive 4 mg TA (*n* = 33) or placebo (*n* = 34). At five years, an improvement in visual acuity of three or more lines occurred in 42% of the eyes in the TA group and 32% of eyes in the placebo group (*P* = 0.4). A worsening of visual acuity by three or more lines occurred in 18% and 24% of eyes, respectively (*P* = 0.88). Mean (±SD) reductions in central macular thickness were 100 ± 79 *μ*m in the TA group and 184 ± 29 *μ*m in the placebo group (*P* = 0.45). After five years, the difference in visual acuity between the two groups was not statistically significant and there was no difference in mean central macular thickness reduction between two groups. Moreover, this study showed that, in the long term, a two-year delay in the beginning of intravitreal TA treatment did not seem to adversely affect outcomes in eyes affected with refractory DME [70].

Novel preservative-free and sustained-release intravitreal implants have been evaluated for the treatment of DME to provide longer duration of pharmacologic effect with lower administration frequency and minimal side effects. I-vation (SurModics, Eden Prairie, MN, USA) is a nonbiodegradable, helical, metal alloy implant coated with polybutyl methacrylate, polyethylene vinyl acetate polymers, and TA. Drug delivery and duration rates can be tuned varying the ratios of the constituent polymers. This system is implanted through a 25-gauge device. A phase I study have shown positive functional and morphological outcomes in 31 patients affected by DME [71]. However, phase IIb trial for I-vation TA was suspended in 2008 following the publication of the DRCR.net study. The Cortiject implant (NOVA63035, Novagali Pharma) is a preservative- and solvent-free emulsion that contains a tissue-activated proprietary corticosteroid prodrug. Once released, the prodrug is activated at the level of the retina. A single intravitreal injection of the emulsion provides sustained release of the corticosteroid over a 6- to 9-month period. An open-label, phase 1, dose-escalation clinical study to assess the safety and tolerability of NOVA63035 in patients with DME is currently underway.

### 2.2. Macular Edema Secondary to Retinal Vein Occlusion

Macular edema is a common cause of reduced vision in patients with retinal vein occlusions. Due to the well-know antiedematous and antipermeability effects, intravitreal TA has been evaluated in many studies on macular edema secondary to CRVO and BRVO. Case series have suggested that intravitreal injection of TA may be useful for the treatment of macular edema in patients with BRVO [72]. However, the use of this pharmacological approach was not supported by the results presented in the Standard Care versus Corticosteroid for Retinal Vein Occlusion (SCORE) Study. In this multicenter clinical trial, 411 participants affected by macular edema secondary to BRVO were randomized to receive laser photocoagulation, 1-mg, or 4-mg doses of preservative-free intravitreal TA (Trivaris). After 12 months of follow-up, the proportion of eyes with an improvement in visual acuity that enabled patients to read 15 or more letters was similar among the three groups (27% in the group treated with the 4-mg dose of TA, 26% in the group treated with the 1-mg dose, and 29% in the control group). Results showed that there was no difference identified in visual acuity at 12 months for the laser group compared with the TA groups. The duration of the edema is an important issue to be considered. Among patients with a duration of macular edema that is more than 3 months, a proportion of 34% of eyes showed a gain of 15 letters or more in the 4-mg TA group, versus a percentage of 15% of patients in the photocoagulation group. However, these findings were not statistically significant but indicated the importance of taking into account the duration of edema in data analysis and in clinical practice [47]. Several clinical trials have also published the beneficial effects of intravitreal administration of TA for the treatment of macular edema due to CRVO [73]. In a 12-month randomized clinical trial, 271 patients affected by macular edema secondary to nonischemic CRVO were randomly assigned to observation, 1-mg or 4-mg doses of preservative-free intravitreal TA (Trivaris). At 1 year, the proportion of eyes with an improvement in visual acuity of 15 or more letters was 26% in the group treated with the 4-mg dose of TA, 27% in the group treated with the 1-mg dose, and 7% in the control group (*P* = 0.001) [46]. Verisome (Icon Bioscience Inc, Sunnyvale, CA, USA) is a biodegradable implant designed to be injected intravitreously and release TA for up to one year.

The Verisome delivery system is a sustained-release drug delivery system that can be injected into the eye as a liquid via a standard 30-gauge needle. When injected into the vitreous, the liquid coalesces into a single spherule. A phase I trial was conducted in patients with macular edema associated with RVO evaluating the drug delivery system at two dosing levels, a 25-*μ*L dose designed to last 6 months, and a 50-*μ*L dose designed to last one year in the vitreous cavity. The promising results of the clinical trial confirmed the safety and efficacy outcomes and the controlled-release attributes of the technology [74].

### 2.3. Pseudophakic Cystoid Macular Edema

Postoperative cystoid macular edema may be a complication of cataract surgery. This condition is typically treated with topical, peribulbar, and systemic administration of steroids and nonsteroidal anti-inflammatory agents. Recently, promising results have been obtained using intravitreal TA for the treatment of this condition [50].

### 2.4. Other Indications

Intravitreal administration of TA has been increasingly performed as an alternative option for the treatment of exudative age-related macular degeneration either in monotherapy or in combination with anti-VEGF drugs. Furthermore, TA has recently been used in combination with pars plana vitrectomy for proliferative diabetic retinopathy and proliferative vitreoretinopathy. Intravitreal TA is also a useful surgical tool for assisting vitreoretinal surgery because besides visualizing the vitreous body, it allows a sharp contrast between the peeled and unpeeled retina, promoting the removal of the membranes that are readily visualized. TA-assisted peeling has been reported during macular hole and macular pucker surgery [75]. Other conditions that can benefit from intravitreal TA are uveitis and immunological disorders, cystoid macular edema after penetrating keratoplasty, and progressive ocular hypotony [76, 77].

## 3. Dexamethasone

Dexamethasone is a potent inhibitor of cytokines released by human pericytes and it has demonstrated high levels in the vitreous for more than 6 months in vivo. Preclinical studies have reported that intravitreal injection of dexamethasone decreases significantly Intercellular Adhesion Molecule-1 (ICAM-1) mRNA, and protein levels, reducing leukostasis and BRB breakdown [78]. Dexamethasone has a relatively short half-life (about 3.5 hours), but is five times more potent than TA [79, 80]. An innovative intravitreal dexamethasone implant has been developed to permit a sustained and extended release of corticosteroids in the intravitreal cavity. A biodegradable dexamethasone drug delivery system (DDS) has been created by Allergan (Ozurdex, Allergan, Irvine, CA, USA). Ozurdex was designed to provide sustained distribution of 700 *μ*g of dexamethasone in the vitreous cavity. The implant is formed by a solid biodegradable polymer (NovadurTM, Allergan, Irvine, CA, USA), whose degradation produces lactic acid and glycolic acid, which are subsequently converted to and eliminated as carbon dioxide and water. The dexamethasone implant is administered as an office-based intravitreal injection using a novel 22-gauge injecting applicator [81]. Recently, Chang-Lin et al. have published pharmacokinetics and pharmacodynamics data of Ozurdex. It was observed that the opaque, round cylindrical implant became translucent, fragmented, and smaller two months after implantation. The concentration of dexamethasone was detected in the retina and vitreous humor for 6 months, with peak concentrations during the first 2 months. Dexamethasone concentrations in the vitreous and in the retina were characterized by two distinct phases, which corresponded to the fragmentation of the implant. On day 60, high levels of dexamethasone were detected in the posterior segment, with the mean peak concentration of 1110 ± 284 ng/g in the retina and 213 ± 49 ng/mL in the vitreous. Following a relatively rapid decline in concentration between day 60 and 90, a second steady state is reached and maintained through day 180 [82].

The Ozurdex dexamethasone-sustained delivery implant has been approved by the United States Food and Drug Administration (FDA) for the treatment of macular edema associated with retinal vein occlusion (RVO) and for noninfectious posterior uveitis.

### 3.1. Macular Edema Secondary to Retinal Vein Occlusion

FDA approval was based on the therapeutic effects of dexamethasone implant investigated in a randomized, controlled clinical trial (the Ozurdex GENEVA study) [83]. The study design included two identical, randomized, prospective, multicenter, masked, and sham-controlled parallel groups. In the double-masked 6-month initial treatment phase, 1.262 eyes were randomized to either a sham procedure (*n* = 426) or treatment with 350 *μ*g (*n* = 414) or 700 *μ*g (*n* = 427) dexamethasone implant. In the second open-label phase, all eligible eyes received a 700 *μ*g dexamethasone implant and were followed-up for additional 6 months. The primary endpoint was the time to achieve over 15-letter improvement (3 Snellen lines) in BCVA, and the secondary outcomes included BCVA over the 6-month trial period and central retinal thickness measured by OCT. The proportion of eyes that achieved an improvement in visual acuity of 15 or more letters was 22% in the 700 *μ*g group, 23% in the 350 *μ*g group, and 13% in the sham group at month 3 (*P* < 0.001). These data were no longer statistically significant at month 6. At the end of the follow-up, the percentage of eyes that had experienced a three-line gain was 41% in the 700 *μ*g group, 40% in the 350 *μ*g group, and 23% in the sham group (*P* < 0.001). The reduction in mean central retinal thickness was greater in the 700 *μ*g (208 ± 201 *μ*m) and 350 *μ*g (177 ± 197 *μ*m) groups than in the sham group (85 ± 173 *μ*m) at month 3 (*P* < 0.001), but not statistically significant at month 6. Twenty-one percent of the eyes affected by BRVO and the 17% of eyes with CRVO required only a single treatment after 12 months of follow-up. The study was also able to show that early treatment of macular edema was more beneficial than delayed treatment in restoring VA. A post hoc analysis suggested that eyes treated within 90 days since the onset of cystoid macular edema were more likely to improve than eyes in which the treatment was instituted after this time point. In addition to being the first FDA-approved therapy for macular edema related to RVO, the dexamethasone DDS has been approved by the EMA for macular edema in eyes with RVO in all of the 27 member states of the European Union.

### 3.2. Pseudophakic Cystoid Macular Edema and Macular Edema Secondary to Uveitis

Cystoid macular edema is a condition that can cause vision impairment after cataract surgery or uveitis. In a randomized, prospective, single-masked, controlled trial, 41 eyes with persistent macular edema from uveitis or Irvine-Gass syndrome were randomized to receive 350 *μ*g, 700 *μ*g dexamethasone DDS, or observation. Results have shown that dexamethasone DDS is significantly effective than observation. An improvement in visual acuity by three or more lines was seen in 53.8% of 700 *μ*g-treated eyes compared with 7.1% of observed eyes (*P* = 0.008). Moreover, 58% of eyes treated with the 700 *μ*g implant have experienced an improvement in angiographic leakage, compared with only 8% of untreated eyes (*P* = 0.027) [79].

### 3.3. Diabetic Macular Edema

The efficacy of dexamethasone DDS was evaluated in a randomized controlled study on patients with persistent macular edema, defined as persistence of macular edema for more than 90 days despite treatment. A clinical trial enrolled 315 eyes with persistent macular edema associated with numerous eye conditions, including DME. One hundred seventy-two diabetic patients were randomized to receive either a 350 or 700 *μ*g implant or observation. At 6 months, a visual acuity gain of at least 2 lines was obtained in 32.4%, 24.3%, and 21% of the eyes receiving the 350-*μ*g implant, the 700-*μ*g implant, and observation, respectively. Treated eyes also had significantly greater improvements in central macular thickness and fluorescein leakage [84].

The CHAMPLAIN study was a prospective, multicenter trial that enrolled adults with DME in a vitrectomized eye. The authors reported that 21.4% of diabetic eyes gained at least 10 letters and 42.9% of eyes had improved at least 5 letters of visual acuity. Central macular thickness was decreased by 27% at week 13 and 9.6% at week 26 after the dexamethasone intravitreal implant [85].

### 3.4. Age-Related Macular Degeneration

Intravitreal dexamethasone is also used in clinical practice as a part of adjuvant therapy to treat exudative AMD. Neovascular AMD is a multifactorial process that involves choroidal neovascularization (CNV), vascular leakage, and inflammation. A triple therapy approach to the treatment of wet AMD can be employed when monthly treatment with vascular endothelial growth factor inhibitors (anti-VEGF) has failed. Triple therapy (TT), traditionally, consists of a vaso-occlusive procedure (photodynamic therapy, PDT), an anti-VEGF agent, and a steroid. The dose of dexamethasone used in numerous TT trials ranges from 200 *μ*g to 800 *μ*g per injection. Several recent studies have shown that TT may reduce the total number of injections of anti-VEGF required and may stabilize vision in those patients not responding to anti-VEGF monotherapies. Augustin et al. investigated the efficacy and safety of TT with PDT-V (42 J/cm^2^), intravitreal dexamethasone (800 *μ*g), and intravitreal bevacizumab (Avastin; Genentech, San Francisco, CA, USA, and Roche, Basel, Switzerland)(1.5 mg). One hundred-four eyes were included in this study. On average, an increase in visual acuity of 1.8 lines was reported after a mean follow up of 40 weeks. Eighteen eyes required an additional intravitreal bevacizumab injection and 5 eyes necessitated a second cycle of TT [86]. Bakri et al. reviewed retrospectively the safety and efficacy of same-day therapy with PDT-V (25 J/ cm^2^), intravitreal dexamethasone (200 *μ*g), and bevacizumab (1.25 mg) in 31 eyes. Visual acuity improved from 0.61 logMAR to 0.58 logMAR after a mean follow-up of 13.7 months. Retreatment was given with a mean of 2.3 anti-VEGF injections and 0.3 repeated TT treatments [87]. Ehmann and García studied prospectively the safety and efficacy of same-day PDT-V (25 J/ cm^2^) and intravitreal dexamethasone (800 *μ*g). At 1 and 7 weeks, patients received a bevacizumab (1.25 mg) injection. Thirty-two eyes were included and then followed-up for 12 months. Visual acuity significantly improved from 0.74 ± 0.33 logMAR to 0.53 ± 0.32 logMAR (*P* < 0.005). The authors reported that a proportion of 31% of the eyes had gained more than 3 lines and a percentage of 6% of the eyes had experienced a loss of more than 3 lines. Central macular thickness was decreased from 328 ± 116 *μ*m to 216 ± 85 *μ*m (*P* < 0.001) at 12 months of follow-up. The mean number of treatment cycles was 1.4, while the mean number of bevacizumab injections was 2.8 at the end of follow up [88].

Randomized controlled studies evaluating the use of the dexamethasone implant in combination with ranibizumab (Lucentis; Genentech Inc, San Francisco, CA, and Novartis AG, Basel, Switzerland) in patients affected by neovascular AMD have found that the implant significantly delayed or reduced the need for repeated ranibizumab injection [89–91]. A 26-week multicenter open-label trial has been conducted to evaluate efficacy and safety of dexamethasone implant in combination with intravitreal ranibizumab in the treatment of naïve subjects affected by subfoveal CNV secondary to AMD. All eyes received the dexamethasone implant at the baseline. From week 2 study visit, eyes were eligible for treatment with ranibizumab 0.5 mg if BCVA had dropped 5 letters or more from baseline. From weeks 4 to 22, ranibizumab 0.5 mg could be given at the physician's discretion. The use of dexamethasone implant alone resulted in statistically significant improvements in CRT from baseline as early as week 1 and continued through week 4 (*P* < 0.001). In addition, clinically significant improvements in BCVA and fluorescein leakage were seen with the implant alone. With the addition of ranibizumab as needed, statistically significant improvements in CRT, BCVA, and FA leakage were more pronounced (*P* < 0.001). The percentage of eyes achieving at least a 15-letter improvement from baseline BCVA was 4.5% at week 4, 11.4% at week 8, 20.5% at week 22, and 15.9% at week 26. Eighty-four percent of the patients did not require rescue treatment with ranibizumab before 4 weeks. By the end of the follow-up, a percentage of 45.5% required 3 or fewer injections of ranibizumab and 20.4% of eyes needed 1 injection or fewer [91].

In the RADICAL Study, 162 patients were randomized to one of four treatment arms: double therapy with reduced fluence PDT-V (25 J/ cm^2^) followed by ranibizumab, reduced-fluence PDT-V (25 J/ cm^2^) followed by ranibizumab-dexamethasone triple therapy, very low-fluence PDT-V (15 J/ cm^2^) followed by ranibizumab-dexamethasone triple therapy, or ranibizumab monotherapy. The 24-month results showed that mean visual acuity change from baseline was not statistically different among the treatment groups. Mean visual acuity in the double therapy group decreased by 2 ETDRS letters, in the TT half-fluence group improved by 2 letters, in the TT very-low fluence group improved by 0.3 letters, and in the monotherapy group improved by 3.8 letters. Through 24 months, patients in the TT half-fluence group had a mean of 4.2 retreatments compared with 8.9 for the ranibizumab monotherapy group. However, when computing the burden of the treatment protocol, the number of individual treatments was 12.6 in the TT half-fluence group and 8.9 in the ranibizumab monotherapy group [92].

### 3.5. Noninfectious Vitritis

The dexamethasone DDS has also proven beneficial in the treatment of noninfectious vitritis, and has been recently approved by FDA and EMA for this ocular condition. In a randomized, 26-week, sham-controlled phase 3 trial, 229 eyes with noninfectious, intermediate, or posterior uveitis were randomized to a single treatment with a 700 *μ*g dexamethasone DDS (*n* = 77), a 350 *μ*g dexamethasone DDS (*n* = 76), or sham injection (*n* = 76). The dexamethasone DDS was significantly more effective than sham in removing vitreous haze. At week 8, a complete resolution of vitreous haze was seen in 47% of 700 *μ*g dexamethasone group, 36% of 350 *μ*g dexamethasone group, and 12% of sham group (*P* < 0.001). This beneficial effect persisted through the end of the study. At all study visits, the proportion of eyes with a gain of 15 or more letters from baseline BCVA was significantly greater in dexamethasone- treated groups than in sham-treated eyes [93].

## 4. Fluocinolone Acetonide

Fluocinolone acetonide is a synthetic corticosteroid with potency similar to the glucocorticoid dexamethasone. It is a corticosteroid with 1/24 the solubility of dexamethasone in aqueous solution, which presumably would allow steroid release over a much longer time period. Pharmacokinetic studies have been conducted on rabbits implanted with 0.5 and 2 mg implants and have found constant levels of fluocinolone acetonide in the vitreous at all time points tested from 2 hours to 12 months postimplantation, indicating zero-order kinetics [94]. Vitreous concentrations were 7-8 times higher in rabbits treated with 2 mg implants compared with those with 0.5 mg implants. Steroid concentrations in the retina and vitreous were considerably higher than those measured in the aqueous humor, indicating posterior localization. Urine and plasma levels of fluocinolone were below the threshold of detection of 200 pg/mL, indicating the lack of systemic absorption. The findings have been confirmed in human trials, where fluocinolone was also undetectable in blood samples [95]. These results reinforce the local activity of the fluocinolone implant and the low risk of systemic adverse effects of corticosteroids. The positive results reported in several pilot, follow-up, and multicentered trials suggest that the fluocinolone acetonide intravitreal implant may play a significant role in the treatment of noninfectious posterior uveitis, providing long-term control of posterior segment inflammation. In addition to a decrease in the recurrence of uveitis, these studies have showed an improvement in visual acuity in implanted eyes and a reduction of the use of combination therapy with systemic steroids or local injections. As the result of clinical trials demonstrating safety and efficacy, the United States FDA approved the 0.59 mg fluocinolone acetonide intravitreal implant (Retisert, Bausch & Lomb, Rochester, NY) as a first choice for the treatment of noninfectious posterior uveitis in April 2005 [96].

### 4.1. Diabetic Macular Edema

Recently, fluocinolone acetonide implants have been studied for the treatment of other ocular conditions, including DME. Iluvien (Alimera Sciences, Alpharetta, GA, USA) has been developed as a nonbiodegradable intravitreal insert for the sustained delivery of fluocinolone acetonide to the posterior segment. It is designed to be injected with a 25-gauge needle through the pars plana. The device is not secured to the sclera but remains free-floating in the vitreous. The Iluvien contains approximately 190 *μ*g of fluocinolone acetonide. Depending on its formulation, the insert can deliver a low dose of approximately 0.2 *μ*g per day, with a delivery lifespan of more than 2 years, or a high dose of approximately 0.5 *μ*g per day, with a lifespan of approximately 18 months. The FAME study consisted of two 36-month phase 3 clinical trials that investigate the safety and the efficacy of two doses of FA implant in patients affected by DME. In this study 956 patients with DME were randomized to either receive a high dose insert (0.5 *μ*g/day), a low-dose insert (0.2 *μ*g/day), or a sham insertion. These trials have reported that 26.8% of low dose FA group and 26.2% of high dose FA group gained 15 or more letters at 24 months compared with 14.7% of patients randomized to control (*P* = 0.029 and *P* = 0.032, resp.). At 36 months, an improvement in visual acuity by three or more lines occurred in 28.7%, 27.8%, and 18.9% of eyes, respectively. Mean (±SD) reductions in central macular thickness were 185 ± 174 *μ*m in the high dose FA group, 180 ± 160 *μ*m in the low dose FA group, and 142 ± 152 *μ*m in the sham group at the end of follow-up period (*P* < 0.001, *P* < 0.005 and *P* < 0.001, resp.). Among patients with a duration of DME that is more than 3 years (long duration), a proportion of eyes showing a gain of 15 letters or more was 13.4% of patients in sham group compared with 34% in the low dose FA group (*P* < 0.001) and 28.8% in high dose FA group (*P* = 0.002). An improvement of three or more lines in patients with DME for less than 3 years (short duration) occurred in 27.8% of the eyes in the sham group, 22.3% of the eyes in low dose FA group, and 26.4% of the eyes in high dose FA group, but the difference was not significant. The mean change in BCVA letter score between baseline and month 36 in long duration DME subjects was 1.8 in the sham group compared with 7.6 in low dose FA group (*P* < 0.004) and 6.2 in high dose FA group (*P* < 0.024). Similar and not significant anatomic outcomes were seen in patients with short a long duration DME [97]. FA implant is still not approved by FDA, but it has recently received marketing authorization in UK, Austria, France, Germany, Spain, and Portugal for the treatment of vision impairment due to chronic DME unresponsive to other available therapies.

### 4.2. Other Conditions

Iluvien insert is currently in phase II clinical trials for the treatment of dry AMD, macular edema secondary to RVO and in several studies comparing 0.2 mg and 0.5 mg fluocinolone acetonide intravitreal insert and ranibizumab in neovascular AMD.

**Figure 1:**
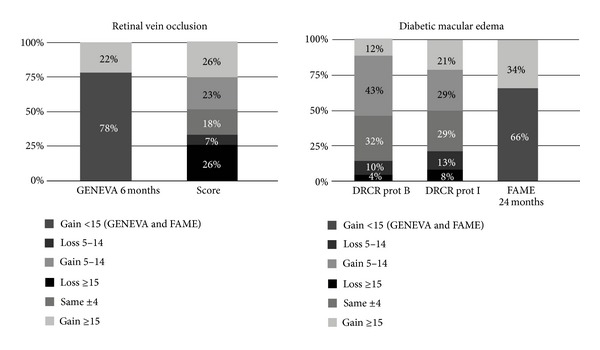
Distribution of change in visual acuity (ETDRS letters) between baseline and month 12 (unless otherwise specified) for large, controlled, and randomized clinical trials investigating steroids in diabetic macular edema and macular edema secondary to retinal vein occlusion. GENEVA (6 months): 700 *µ*g dexamethasone implant. SCORE: 4 mg intravitreal triamcinolone acetonide. DRCR prot B: 4 mg intravitreal triamcinolone acetonide. DRCR prot I: 4 mg intravitreal triamcinolone acetonide plus laser photocoagulation. FAME (24 months): 0.2 *μ*g/day fluocinolone implant. GENEVA and FAME publications did not disclose distribution of change other than the percentage of patients showing a ≥ 15 ETDRS letters gain.

A summary of change in visual acuity from studies investigating steroids in DME and macular edema secondary to RVO is presented in Figure 1.

## 5. Safety of Intravitreal Corticosteroids

### 5.1. Triamcinolone Acetonide

Potential complications of intravitreal corticosteroid treatment are divided into steroid-related and injection-related adverse effects. Steroid-related side effects most commonly include cataract formation and an intraocular pressure (IOP) increase. Injection-related side effects include retinal detachment, endophthalmitis, and pseudoendophthalmitis.

#### 5.1.1. Postinjection Infectious Endophthalmitis

Infectious endophthalmitis is one of the most serious complications of intravitreal injection of TA, with the reported risk per injection ranging from 0.1% to 1.6% [20]. Many studies suggest that this relatively high rate of infectious endophthalmitis may be attributed to the techniques used for injection. If the injection is performed under sterile conditions, the risk of an infection may be inferior [98, 99].

#### 5.1.2. Postinjection Pseudoendophthalmitis

Several studies have described noninfectious endophthalmitis after intravitreal injection of TA [98, 99]. Postinjection pseudoendophthalmitis is present if TA crystals are washed from the vitreous cavity into the anterior chamber and settled down in the inferior anterior chamber angle mimicking a hypopyon. According to reports, this complication occurs in 0.2%–6.7% of the eyes following treatment. TA crystals in the anterior chamber usually disappear spontaneously and may not need to be removed. There have been no reports so far showing corneal endothelial damage or damage to the trabecular meshwork by the crystals [100]. Some investigators have hypothesized that the presence of benzyl alcohol, a bacteriostatic preservative in some commercially available TA, leads to an increased risk of sterile endophthalmitis [101].

#### 5.1.3. Postinjection Ocular Hypertension

A number of reports have described intraocular pressure (IOP) elevation as the most common adverse event of intravitreal TA [102, 103]. Mild to moderate IOP elevation was seen in 28%–42% of patients, typically within the first 3 months following injection. This condition is usually controlled with topical agents alone. About 1% of the patient requires surgical treatment. Selective laser trabeculoplasty (SLT) is a treatment alternative or adjunct to medical therapy. Comparing studies using different doses of TA for intravitreal injection may suggest that the risk of IOP rise appears to be higher due to the prolonged elevated concentrations that are achieved intraocularly. If further studies confirm the assumption that the frequency of secondary ocular hypertension after an intravitreal TA injection may not markedly depend on the dose used, one may assume that even relatively low TA doses are already high enough to occupy all steroid receptors. Some authors suggest that a premedication with topical steroids may be useful to identify possible steroid-responders and excluding those from intravitreal TA treatment that may lower the incidence of IOP elevation [104].

#### 5.1.4. Post Injection Cataract

Steroid-induced cataract is a common side effect of intravitreal TA. A recent study reported that in the elderly population intravitreal, high-dose injections of TA lead to clinically significant cataract with eventual cataract surgery in about 15–20% of the eyes within about one year after the intravitreal injection [105]. Jonas et al. concluded that eyes with an elevation of IOP after intravitreal TA have a very high risk of rapidly experiencing posterior subcapsular lens opacities [106]. This strong association suggests a similar mechanism responsible for the development of steroid-induced posterior subcapsular cataract and for the elevation of IOP. A study suggested that a single intravitreal TA induces posterior subcapsular cataract development, whereas multiple injections result in all-layer cataract progression [107].

#### 5.1.5. Rhegmatogenous Retinal Detachment

A potential complication of the intravitreal TA injection may be a rhegmatogenous retinal detachment [108]. Triamcinolone acetonide, injected into the vitreous cavity, leads to a change in the structure of the vitreous body and the abnormal vitreous may exert traction on the retina. In particular, this is supposed for the inferior midperipheral area of the vitreoretinal interface where the TA crystals remain in the preretinal vitreal cortex, for superior midperipheral and peripheral regions where a vitreous traction might be induced by the weight of the TA crystals settled at 6 o' clock and for the far periphery of the fundus where the vitreous, incarcerated into the injection site, causes retinal traction.

#### 5.1.6. Toxic Effects

Previous studies in rabbit found that preservatives in the vehicle for suspension of crystalline steroid, rather than steroid itself, could be toxic to the rabbit retina and lens and that the vehicle is not totally responsible for the toxicity, but may initiate TA-dependent toxicity [109]. Direct toxic effects of TA on the retina and optic nerve have not yet been observed, independently of the dose used. Triamcinolone acetonide has been shown to be toxic to retinal pigment epithelial cells in vitro [110], whereas ex vivo [111] and in vivo [112] studies have failed to show any significant toxicity to the retina. Because TA is a heavy depot formulated suspension, it settles in the inferior vitreous cavity. Whereas there is certainly distribution of the drug throughout the vitreous cavity due to diffusion and constant eye movements, it is possible that the drug does not distribute equally in the vitreous cavity and that the concentration of the drug at the macula is different (presumably lower) than in the inferior retinal periphery [113]. Yeung et al. reported a possible cytotoxic effect of TA, causing a significant reduction in cell numbers throughout the whole range of concentrations when retinal pigment epithelium cells were exposed to it for more than one day [114].

#### 5.1.7. Systemic Safety

In the randomized study from DRCR.net, comparing laser photocoagulation to ranibizumab in combination with laser and intravitreal TA associated with laser, no evidence suggest that the administration of TA is associated with an increased risk of systemic adverse events, including stroke or cardiac events. Two-year incidence of nonfatal myocardial infarction was 3% in the laser alone group, 1% in the ranibizumab-laser group, and 3% in the TA-laser group. Any cardiovascular event, as defined by Antiplatelet Trialists' Collaboration (ATC), occurred in the 12% in the laser alone group, 5% in the ranibizumab-laser group, and 6% in the TA-laser group [61]. Reports of systemic adverse events were similar between the SCORE-BRVO and CRVO trial groups. The medical dictionary for regulatory activities system/organ class of infection and infestations had the highest incidence through month 12, with 10%, 15-16%, and 15–19% of participants reporting at least 1 event in the standard care, 1-mg TA, and 4-mg TA groups, respectively [46, 47].

### 5.2. Dexamethasone

The safety and tolerability of a sustained-release implant are particularly important due to the long duration of exposure to the drug and the drug vehicle. The safety of the implant may be divided into several categories: complications arising from the implantation procedure; toxicity or immunoreactivity associated with exposure to the implant polymer; and ocular adverse events associated with exposure to the agent itself, such as cataract formation and IOP increase.

#### 5.2.1. Traumatic Implantation

Several adverse events were believed to be related to traumatic implantation. A recent study compared the safety profile of surgical implantation with that of a novel proprietary applicator device. Use of the applicator device was associated with a lower overall incidence of ocular adverse events (68.4% versus 90%), although this difference was not statistically significant. Of note, there were no reports of vitreous hemorrhage in the applicator group, compared with 2 out of 10 patients in the incisional group who experienced this complication. However, the study was insufficiently powered to determine a statistically significant difference for this or any other infrequently occurring adverse event [115].

#### 5.2.2. Toxic Effects

Early animal studies determined that a high concentration of dexamethasone could be achieved intravitreally without any clinical, histological, or electrophysiological toxicity [80]. Increasing levels of retinal toxicity have been reported at doses above 800 *μ*g administered to rabbits. Electroretinographic studies confirmed no change in normal retinal physiology [80].

#### 5.2.3. Postimplant Ocular Hypertension

A number of studies have described intraocular pressure (IOP) elevation as a common adverse event of dexamethasone implant. Kuppermann et al. reported that an elevation of IOP more than 10 mmHg occurred in less than 20% of each groups treated with dexamethasone implant. This condition is usually controlled with topical agents alone [84]. The recent data presented by GENEVA study have shown transient instances of elevated IOP, which were managed either by observation or with topical medications alone. Typically, IOP reached a peak at 2 months, decreasing steadily over the next 4 months. At this peak time point, the authors reported that 16% of all patients had a pressure of greater than 25 mmHg. The proportion of patients using ocular antihypertensive agents increased from 6% at study entry to 24% at 6 months among all patients in the treatment group. An increase of at least 10 mmHg from baseline was seen in 12.6% of study eyes at 2 months after the first dexamethasone implant and 15.4% of study eyes at 2 months after the second dexamethasone implant. At the end of the 12-month study period, 32.8% of retreated patients had at least a 10 mmHg increase from baseline and 14 eyes required laser or surgery to reduce intraocular pressure [83].

#### 5.2.4. Postimplant Cataract Formation

Comparing patients treated with 0.35 mg or 0.7 mg doses of dexamethasone implant with a control group, many randomized clinical trial have recorded that the rate of cataract formation was not significantly different in any treatment group than in the control group at the end of 6 months of follow-up [83, 84]. However, GENEVA study reported that after 12 months of follow-up, cataracts were listed in 29.8% of phakic study eyes in the retreated DEX 0.7/0.7 group, 19.8% of the DEX 0.35/0.7 group, and 10.5% of the delayed treatment (sham/0.7) group (*P* = 0.001) [83].

#### 5.2.5. Fluocinolone Acetonide

Randomized clinical trials evaluating the safety of Retisert for the treatment of uveitis have reported that a proportion from 50% to 90% of patients experienced an adverse event after implantation, most commonly cataract formation and increased IOP. Within 2 years of implantation, nearly 100% of phakic eyes required cataract surgery and one third of patients required a glaucoma surgical procedure. Other adverse events included ptosis, eyelid edema, conjunctival hemorrhage, chemosis, corneal edema, vitreous opacities, vitreous hemorrhage, macular edema, retinal hemorrhage, hypotony, and choroidal detachment [95, 96].

In the randomized study from FAME comparing sham injection, high-dose and low-dose of fluocinolone implant presented two ocular adverse events: cataract progression and intraocular pressure (IOP) increase. The most common adverse event was cataract, which was listed in 42.7% of the low-dose group, 51.7% of the high-dose group, and 9.7% of the sham group. Of those patients who were phakic at baseline, cataract surgery was performed in 80.0% (low dose) and 87.2% (high dose) of patients in the fluocinolone groups compared with 27.3% in the sham group. During the study elevation of IOP, more than 30 mmHg was recorded in 37.1% of patients in the low-dose group, 45.5% in the high-dose group, and 11.9% in the sham group. Laser trabeculoplasty was performed in 2.5% of the high-dose group, 1.3% of the low-dose group, and 0% of the sham group. Incisional IOP-lowering surgery was done in 8.1% of the high-dose group, 4.8% of the low-dose group, and 0.5% of the sham group [97].

## 6. Conclusions

Intravitreal steroid injection appears to be an effective option for the treatment of macular edema secondary to various etiologies. The rationale for using steroids is due to anti-inflammatory, antiedematous, and antiangiogenic properties. An increasing number of ophthalmologists use intravitreal steroids for the treatment of various posterior segment disorders, especially when traditional therapeutic methods have failed. Triamcinolone acetonide is a drug that has largely been studied in many clinical trials for the treatment of these ocular conditions. However, the need for frequent intravitreal injections and the potential side effects have focused attention on the development of alternative systems for the delivery of ophthalmic medications. A variety of methods have been proposed that achieve longer duration of pharmacologic effect with lower administration frequency and minimal side effects. Novel agents including preservative-free and sustained-release intravitreal implants such as Ozurdex and Iluvien are currently approved for ocular use and are being further evaluated for the treatment of RVO, DME, uveitis, and AMD. Due to a potential for greater potency, dexamethasone and fluocinolone acetonide are being evaluated alone or in combination with anti-VEGFs as promising options in the emerging armamentarium for the treatment of several retinal diseases.
